# The Proangiogenic Effect of Iroquois Homeobox Transcription Factor Irx3 in Human Microvascular Endothelial Cells*[Fn FN2]

**DOI:** 10.1074/jbc.M114.601146

**Published:** 2014-12-15

**Authors:** Kisha Scarlett, Vaishnavi Pattabiraman, Petrina Barnett, Dong Liu, Leonard M. Anderson

**Affiliations:** From the ‡ Cardiovascular Research Institute and; ¶ Department of Physiology, Morehouse School of Medicine, Atlanta, Georgia 30310 and; the § Cancer Center for Therapeutic Development, Clark Atlanta University, Atlanta, Georgia 30314

**Keywords:** Angiogenesis, Cell Migration, Endothelial Cell, Notch Pathway, Transcription Factor, VEGF, Irx3

## Abstract

Angiogenesis is a dynamic process required for embryonic development. However, postnatal vascular growth is characteristic of multiple disease states. Despite insights into the multistep process in which adhesion molecules, extracellular matrix proteins, growth factors, and their receptors work in concert to form new vessels from the preexisting vasculature, there remains a lack of insight of the nuclear transcriptional mechanisms that occur within endothelial cells (ECs) in response to VEGF. Iroquois homeobox gene 3 (*Irx3*) is a transcription factor of the Iroquois family of homeobox genes. Irx homeodomain transcription factors are involved in the patterning and development of several tissues. *Irx3* is known for its role during embryogenesis in multiple organisms. However, the expression and function of *Irx3* in human postnatal vasculature remains to be investigated. Here we show that *Irx3* is expressed in human microvascular endothelial cells, and expression is elevated by VEGF stimulation. Genetic *Irx3* gain and loss of function studies in human microvascular endothelial cells resulted in the modulation of EC migration during wound healing, chemotaxis and invasion, and tubulogenesis. Additionally, we observed increased delta-like ligand 4 (*Dll4*) expression, which suggests an increase in EC tip cell population. Finally, siRNA screening studies revealed that transient knockdown of *Hey1*, a downstream Notch signaling mediator, resulted in increased *Irx3* expression in response to VEGF treatment. Strategies to pharmacologically regulate Irx3 function in adult endothelial cells may provide new therapies for angiogenesis.

## Teaser

**Background:** Transcription regulation is essential for angiogenesis, but the role of *Irx3* in this process remains to be defined.

**Results:** *Irx3* promotes endothelial cell migration and tip cell specification through VEGF-Notch signaling.

**Conclusion:** *Irx3* is a novel proangiogenic mediator of endothelial cell migration and cell fate.

**Significance:** Manipulation of *Irx3* may provide novel therapeutic strategies in adult vascular pathologies.

## Introduction

Angiogenesis is a critical component of development, wound healing, and menstruation (1). However, aberrant angiogenesis occurs in several pathological biological processes such as cancer, atherosclerosis, diabetic retinopathy, and rheumatoid arthritis (2). Angiogenic sprouting is initiated in response to an extracellular VEGF ligand gradient, resulting in specification of a leading EC^2^ “tip” cell, characterized morphologically by multiple filopodial extensions, and “stalk” cells that trail behind the tip cell, maintaining connectivity with the preexisting vessel (3). Dll4/Notch signaling regulates the tip *versus* stalk cell phenotype through a mechanism of lateral inhibition, which is a critical element of control in angiogenesis (4). Previous reports have demonstrated that tip cells express increased levels of DLL4 and VEGFR2, whereas stalk cells express higher levels of Notch and VEGFR1 (5). However the molecular mechanisms that completely govern the specification process remain to be fully elucidated.

There are several families of transcription factors that have been implicated in angiogenesis regulation. The E26 transformation-specific family of transcription factors has been shown to regulate angiogenesis by interacting with the VE-cadherin promoter, which is required for the maintenance of the EC monolayer, EC permeability, and proliferation (6). Overexpression of Krüppel-like factors has been demonstrated to block VEGF-mediated angiogenesis through VEGFR-2 (7). Hairy-related transcription factors (HEY/HESR) also play a critical role in angiogenesis (8, 9).

Notch receptor regulation of *Hey1* during to specific EC tip *versus* stalk cell fate promotes productive VEGFR2-mediated angiogenesis *in vivo* (10). Numerous studies have also shown that the Forkhead Box subclass of forkhead transcription factors is required for angiogenesis (7). Foxo1 and Foxo3a have been shown to regulate non-redundant but overlapping genes such as eNOS and Ang2 that are required for postnatal vascularization (11). Recently, through gain and loss of function studies, the ubiquitously expressed NF-E2-related factor (Nrf2) has been shown to promote vascular branching and density through suppression of Dll4/Notch signaling *in vivo* (4). Furthermore, conditional knockout of Nrf2 in the mouse retina revealed a decreased number of tip cells, filopodial extensions, and branch points as well as aberrant activation of Dll4/Notch signaling.

IRX3 is a member of the Iroquois family of three amino acid loop extension class homeobox genes that are evolutionarily highly conserved among species. In humans, Irx genes reside in two clusters of three genes each that encode transcription factors that recognize the unique palindromic DNA binding motif 5′-*ACAnnTGT*-3′ (12–14). Irx genes are essential in early patterning of many embryonic tissues in a spatially and temporally restricted manner (15). *Irx3* specifically is expressed in the neural tube and lateral mesoderm of the chick, mouse, and zebrafish (12); the branching lung endothelium of the developing rat embryo; and in the trabeculated regions of the ventricular chambers of the developing mouse heart (13, 15, 16). The *Irx3* and *Irx5 Drosophila* orthologs araucan and caupolican are essential for the differentiation of wing vein endothelial cells and the formation of the wing veins L1, L3, and L5 (17). Although *Irx3* has been shown to be required for multiple aspects of embryonic patterning and development, including vein development, very little is known about the regulatory mechanisms that control *Irx3* expression in these tissues. Recent reports of IRX function in adult pathophysiological tissues indicate a broader role of IRX gene function than surmised previously (18–20).

Here we report, for the first time, that *Irx3* regulates critical functions for proper angiogenesis *in vitro* in response to the proangiogenic ligand VEGF. *Irx3* genetic loss and gain of function approaches indicate that *Irx3* promotes EC migration during wound healing, EC migration in response to a chemotactic gradient, and tube-like structure network formation in Matrigel assays. We identified the Notch signaling downstream mediator HEY1 as a negative regulator of *Irx3* in response to VEGF. ChIP studies confirm that HEY1 binds to a distal and a proximal site on the *Irx3* promoter, suggesting an inhibition of EC tip cell phenotypic specification. Taken together, these results indicate that *Irx3* is an essential mediator of HMVEC migration as a downstream target of Notch-CBF1-HEY1 signaling to promote EC tip cell specification in response to VEGF. Therefore, *Irx3* may be a useful and novel target for the development of proangiogenic and antiangiogenic therapies in adult vascular pathologies.

## EXPERIMENTAL PROCEDURES

### 

#### 

##### Human Microvascular Endothelial Cell Culture

HMVECs were maintained in EGM-2MV (endothelial growth medium) BulletKit medium (Lonza). Prior to treatment with VEGF-A_165_ (R&D Systems), cells were made quiescent by incubation in EBM-2MV (Lonza) for 12 h. Cells were treated with 20 ng/ml of VEGF in EBM-2MV + 0.4% FBS unless indicated otherwise. Cells were cultured at 37 °C in a 5% CO_2_ incubator and used at passages 3–8 for all experiments.

##### Recombinant Adenovirus Vector Construction

The recombinant adenovirus vectors containing either the human full-length *Irx3* gene cDNA sequence (NM_024336.2) fused to a V5 epitope (Ad.CMV.*Irx3*-v5), the empty control vector (Ad.CMV.Xnull-v5), a synthetic miRNA targeting *Irx3* mRNA with a bicistronic IRES-eGFP reporter (Ad.CMV.*mirIrx3-eGFP*), and a negative control miRNA-IRES-eGFP (Ad.CMV.*mirNeg-eGFP*) were generated using the cloning system according to the protocol of the manufacturer (Invitrogen). Viral adenovirus gateway plasmids were transduced and amplified in HEK293A cells (Invitrogen). Replication-deficient adenovirus particles were purified and titered using the Adeno-X rapid purification and titer kits, respectively, according to the protocol of the manufacturer (Clontech). All constructs are under the control of the human CMV major immediate-early promoter.

##### Growth of Viral Stocks

Adenovirus vectors were amplified in HEK293A cells in minimum Eagle's medium α supplemented with 10% FBS (Invitrogen). HEK293A cells were transduced with the indicated adenoviral vector (m.o.i. = 1) and harvested by centrifugation at the time of 80% cytopathic effect. The virus was released from HEK293A cells by three cycles of freeze-thawing. Cell debris was pelleted by centrifugation, and the supernatant was titered and stored at −80 °C.

##### Recombinant Adenoviral Vector Transduction

Prior to transduction, HMVECs were grown to the indicated confluency in complete EGM-2MV BulletKit medium. On the day of transduction, the medium was removed, and cells were rinsed with PBS (Invitrogen). Cells were then transduced with the indicated adenoviral vector (m.o.i. = 20) for 6 h in 5 ml of EGM serum-free medium. Afterward, the medium was replaced with medium with 5% serum and incubated further overnight at 37 °C in a 5% CO_2_ incubator. Transduced HMVECs were used the day after transduction at the indicated times for all experiments.

##### Quantitative Real-time PCR Analysis

PCR for mRNA abundance analysis was performed as described previously (21). Briefly, total RNA was isolated from HMVECs using the RNAeasy mini kit (Qiagen). RNA (2 μg) was reverse-transcribed with oligo(dT) primers using EcoDry cDNA premix according to the protocol of the manufacturer (Clontech). Quantitative real-time PCR was performed using the LightCycler FastStart DNA Master SYBR Green I master kit and a LightCycler480 real-time thermal cycler (Roche Applied Science). The human primer sequences utilized were as follows: *Irx3*, 5′-ctctccctgctgggctct-3′ (forward) and 5′-caaggcactacagcgatctg-3′ (reverse); *Hey1*, 5′-cgagctggacgagaccat-3′ (forward) and 5′-ggaacctagagccgaactca-3′ (reverse); *Vegfr-2*, 5′-gaacatttgggaaatctcttgc-3′ (forward) and 5′-cggaagaacaatgtagtctttgc-3′ (reverse); and *18s*, 5′-ggaagggcaccaccaggagt-3′ (forward) and 5′-tgcagccccggacattctaag-3′ (reverse). Quantification was performed by comparative Ct method. All PCR product sizes (60–120bp) were analyzed for a single amplicon product using Roche melt-curve analysis software and confirmed on 4% agarose E-gels (Invitrogen).

##### In Situ Hybridization Assays

RNA *in situ* hybridization was performed as described previously (22). HMVECs were cultured on poly-l-lysine-coated coverslips in a 24-well cell culture plate. HMVECs were fixed using a 4% paraformaldehyde solution. After fixation, cells were permeabilized with a premade detergent solution, followed by protease digestion for 25 min at a working concentration of 1:4000. Cells were then incubated with *Irx3* (VA1-13572-01, type 1 550-nm probe) and *Cdh5/VE-Cadherin* (VA4-10782-01, type 4 488-nm probe) mRNA probe sets for 3 h at 40 °C. After the 3-h incubation step, a preamplifier mix and a working amplifier mix were added, and, finally, incubation with a labeled probe mix was performed according to the protocol of the manufacturers (QuantiGene ViewRNA ISH cell assay, Affymetrix). Cells were visualized by confocal microscopy and software (Leica).

##### RNA in Situ Hybridization of Rat Carotid Artery Sections

All procedures and care were approved by the Institutional Animal Care and Use Committee of the Atlanta University Center. We euthanized male Sprague-Dawley rats (350–400 g) with CO_2_ and performed thoracotomies as described previously (23). Animals were perfused with PBS, and vascular tissues were collected for RNA *in situ* hybridization (QuantiGene ViewRNA ISH Tissue 2-plex assay, Affymetrix) using a rat type 1 *Irx3* probe (NM_001107413) and rat type 6 *Pecam1* probe (NM_031591)) according to the protocol of the manufacturer. Labeled sections were viewed using confocal microscopy and software (Leica).

##### Wound Healing Assays

Wound healing assays were performed as described previously (24). HMVECs were seeded at a high density (1 × 10^6^ cells/ml) in each well of a 6-well culture plate and allowed to adhere overnight. Cells were then serum-deprived in endothelial basic medium 2 (EBM2) + 0.5% FBS media overnight. The next day, cells were transduced with the indicated adenovirus vector (m.o.i. = 20) as described previously. The following day, a scratch wound was made across each well with a micropipette tip. Non-adherent cells were removed by washing each well with PBS. Basal medium with VEGF (EBM-2MV, 0.4% FBS, and 20 ng/ml VEGF) was then added back into the wells. Wound closure was monitored over a 12-h period by phase contrast and fluorescent microscopy every 3 h. The results were compared as total wound area at 0 h *versus* the indicated times. Data are expressed as percent wound area *versus* 0 h. The wound area was quantified using image analysis software (ImagePro). Experiments were performed at least six times with at least three replicate wells per experiment.

##### Transwell Migration Assay

Transwell migration assays were performed as described previously (24) with minor modifications. HMVECs were grown under normal growth conditions to 80% confluence. Cells were transduced as described previously (m.o.i. = 20) with the indicated adenoviral vectors and incubated overnight. The next day, transduced cells were serum-deprived for 12 h by replacing normal growth medium with EBM-2MV. Following serum deprivation, HMVECs were pelleted and resuspended at a density of 4 × 10^5^ cells/ml in EBM-2MV medium supplemented with 0.4% FBS seeded in the upper well of a permeable cell membrane (BD Biocoat, Angiogenesis Fluoroblok system, BD Biosciences). EBM-2MV medium supplemented with 0.4% FBS containing 20 ng/ml VEGF was placed in the lower chamber of each well. Cells were incubated for 20 h at 37 °C in a 5% CO_2_ incubator, and then they were fluorescently labeled with CalceinAM fluorescent dye (Corning Life Sciences). Total fluorescence per well was calculated using ImagePro software. Experiments were performed in triplicate and repeated at least five times.

##### Tubulogenesis Assay

HMVECs were grown to 80% confluency (1 × 10^6^ cells/ml) in 10-cm dishes. Cells were then transduced as described previously (m.o.i. = 20). The following day, transduced cells were serum-deprived by replacing complete growth medium with EBM-2MV for 12 h. Next, cells were resuspended in either EBM-2MV medium supplemented with 0.4% FBS or EBM-2MV medium supplemented with 0.4% FBS containing 20 ng/ml VEGF. For VEGF signaling inhibition, cells were preincubated with SU1498 (25 μm) for 2 h in EMB-2MV before seeding. Cells were seeded at a density of 2 × 10^4^ cells/, BD Biosciences). The plates were incubated for 18 h in a 37 °C incubator with 5% CO_2_. After incubation, cells were stained with CalceinAM, and images were captured using fluorescent microscopy (Olympus). Tube-like network structures were analyzed using AngioTool analysis software (25, 26). All treatments were performed in triplicate and repeated at least five times.

##### Fluorescence Microcytometry

HMVECs were transduced by the indicated adenovirus vector, as described previously, in basal growth medium for 12 h. Next, multiple wounds were made across the surface of each plate using the CellComb scratch assay system (EMD-Millipore). Cells were then treated with EBM-2MV, 0.4% FBS, and 20 ng/ml of VEGF or vehicle for 12 h. Following VEGF treatment, cells were harvested, washed with PBS, and incubated overnight at 4 °C with an anti-Dll4 mouse monoclonal antibody or mouse anti-IgG2b isotype control (Abcam, Cambridge, MA). The following day, cells were washed with PBS and incubated with an Alexa Fluor 488 donkey anti-mouse secondary antibody (Invitrogen), and the green fluorescence cell population (events = 3 × 10,000/well) was acquired as described previously (21) using the Guava EasyCyte system (Millipore) Data were analyzed and compared with the isotype control antibody using FlowJo software (Treestar).

##### High-throughput siRNA Screen

A high-throughput siRNA screen (Biology-on-Array, SABiosciences) of 96 transcription factors was performed. HMVECs were reverse-transfected in a 96-well siRNA plate for 6 h in normal growth medium and then made quiescent in EBM-2MV for 12 h. Following quiescence, cells were treated with EBM-2MV, 0.4% FBS, and 20 ng/ml VEGF for 12 h. Total RNA isolation was performed using a 96-well RNA isolation system (Qiagen). RNA was reverse-transcribed using the RNeasy-96 kit (Qiagen). Quantitative RT-PCR for the *Irx3*, *Hey1*, and *18s* genes (see primer sequences above) was performed using the LC480 Lightcycler thermocycler (Roche). Data were analyzed using the provided array analysis software template for all Biology-on-Array siRNA plates using comparative ^ΔΔ^Ct analysis (SABiosciences). Data are expressed as -fold change in transcript abundance *versus* VEGF-treated negative control siRNA wells.

##### ChIP Assays

Protein-DNA immunoprecipitation was performed as described previously (27, 28) and according to the protocol of the manufacturer (Active Motif), but shearing was performed using the S220 sonicator (Covaris) and a low cell SDS shearing buffer kit. Sheared chromatin was incubated with protein G magnetic beads and either a HEY1 monoclonal antibody or IgG2a isotype control at 4 °C overnight. Protein-DNA complexes were eluted from magnetic beads, and cross-links were reversed using proteinase K. Precipitated DNA was then amplified using *Hey1* primers for the distal *Hey1* binding site (5′-acacgatactccccgcgacctttcc-3′ (forward) and 5′-cctaagtgtccacaacttttggctgc-3′ (reverse)), the proximal *Hey1* binding site (5′-ctacggggcgcaagccttcctctc-3′ (forward) and 5′-acttagaggaggggcgaaggaagcg-3′ (reverse)), and the intronic negative *Hey1* control (5′-ccaacacacacatctacaactgg-3′ (forward) and 5′-ccttctctctctgtcatcatcgttttctcc-3′ (reverse)). ChIP PCR data are expressed as -fold enrichment *versus* IgG chromatin input using comparative ^ΔΔ^Ct analysis. Experiments were performed in triplicate at least five times.

##### Statistics

All samples were prepared in a minimum of triplicates. Results from the quantitative analysis were expressed as mean ± S.D.) of at least three independent experiments. Statistical analyses were performed by analysis of variance, and comparisons between groups were performed using Student's *t* test. Differences were considered significant when *p* < 0.05.

## RESULTS

### 

#### 

##### Irx3 Expression Increases in Response to VEGF Treatment

**FIGURE 1.**
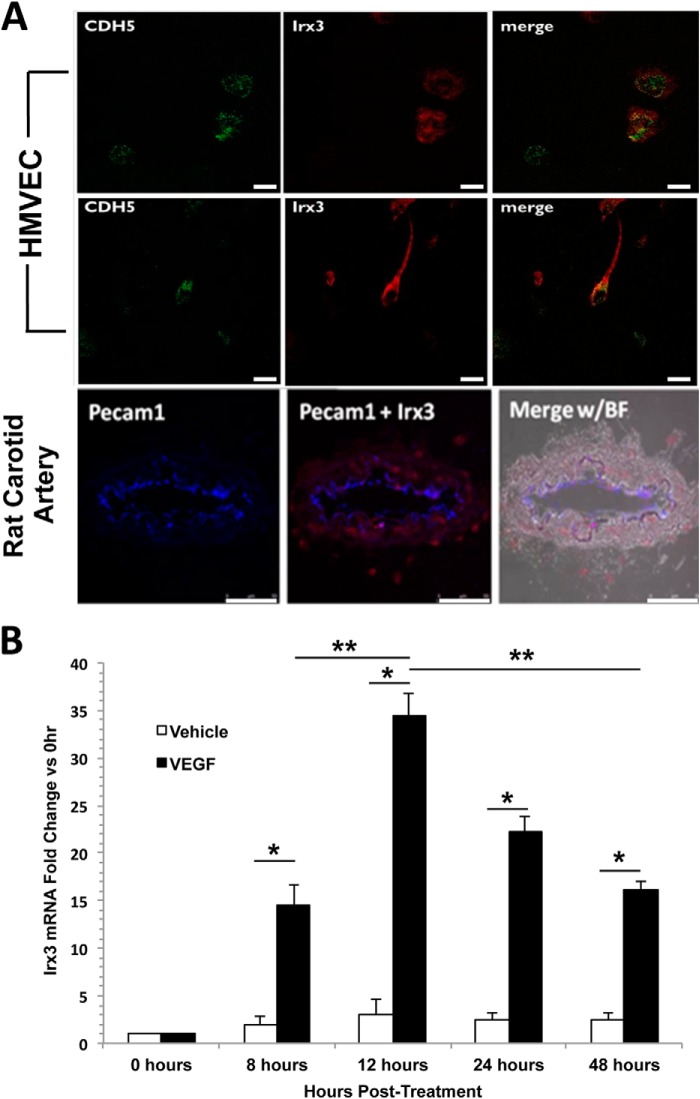
**Irx3 is expressed in microvascular endothelial cells and is stimulated by VEGF-A.** *A*, fluorescence confocal images of *in situ* hybridization of VE-Cadherin (*Cdh5*) and *Irx3* mRNA in HMVECs treated with VEGF-A (20 ng/ml) for 12 h and *in situ* hybridization of *Pecam1* and *Irx3* in adult rat carotid arteries. *Scale bars* = 10 μm (HMVEC) and 50 μm (carotid artery). *B*, HMVECs were grown in basal medium supplemented with 20 ng/ml VEGF-A or vehicle for the indicated times. Total RNA was isolated, and relative mRNA expression levels of *Irx3* were analyzed using quantitative RT-PCR. 18 S rRNA was used as an internal control. Results were compared with corresponding *Irx3* expression levels with time-matched controls and *Irx3* temporal expression levels in response to VEGF-A (*n* ≥ 3). *, *p* < 0.05; **, *p* < 0.01.

To determine whether *Irx3* is expressed in HMVECs *in vitro*, we performed *in situ* hybridization using probes for human *Irx3* and *VE-Cadherin* (*Cdh5*) 12 h after VEGF treatment. Our results indicate that *Irx3* mRNA was distributed throughout the nucleus and cytoplasm of HMVECs (Fig. 1*A*, *top* and *center rows*). Likewise, in cells that have an EC tip-like phenotype, characterized by elongated cell filopodial extensions, *Irx3* expression was observed in the cell periphery and filopodial extension. Next, we investigated *Irx3* expression *in vivo* by performing *in situ* hybridization on tissue sections isolated from rat carotid arteries (Fig. 1*A*, *bottom row*). The results demonstrate that *Irx3* is expressed in endothelial cells lining the lumen of the artery, and expression colocalizes with the EC marker *Pecam1*. Interestingly, *Irx3* expression is also observed in the medial layer of the artery, which also indicates *Irx3* expression present in vascular smooth muscle cells. These data indicate that *Irx3* mRNA is present in endothelial cells *in vitro* and that it colocalizes with the endothelial cell marker *Pecam1* in rat carotid arteries *in vivo*.

To determine the effect of VEGF treatment on *Irx3* gene expression, we treated HMVECs with VEGF over a period of 48 h and then isolated total RNA at various time points for quantitative RT-PCR (Fig. 1*B*). The results indicate that, as early as 8 h, *Irx3* expression increased significantly 14.4-fold (*p* = 0.036) post-VEGF treatment. 12 h post-VEGF treatment, we observed a dramatic increase in *Irx3* expression compared with 0 h (33.8-fold, *p* = 0.006) and 8 h (2.35-fold, *p* = 0.041). Irx3 expression increased in a temporal manner from 8–48 h post-VEGF treatment compared with time-matched vehicle controls. These results also show a reduction in *Irx3* expression after 48 h, which is likely due to cells reaching confluency. These results demonstrate that endogenous *Irx3* expression is elevated in HMVECs in response to VEGF treatment in a temporal manner and peaks in expression at 12 h. Furthermore, *Irx3* was decreased as HMVECs reached confluency 48 h post-VEGF treatment.

##### Gain and Loss of Function of Irx3 Modulates the Endothelial Cell Migratory Phenotype

**FIGURE 2.**
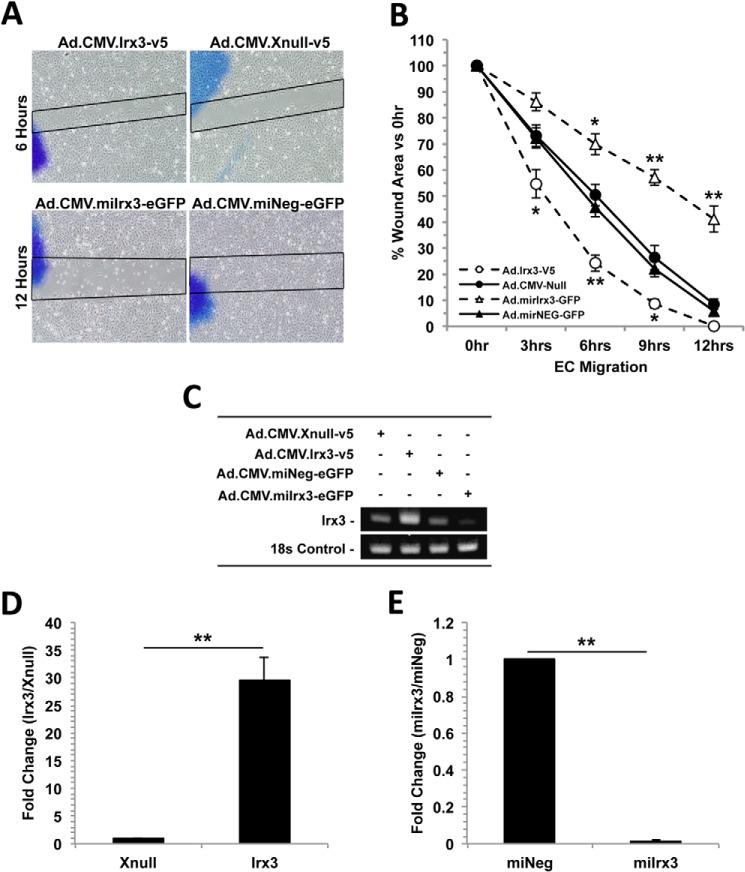
**Irx3 promotes HMVEC migration during wound healing.** Recombinant adenoviral vectors containing gain of function (CMV-IE promoter-*hIrx3-v5*-poly(A) or the control vector containing the CMV-IE promoter-*v5*-poly(A)) or loss of function (CMV-IE-*miIrx3-eGFP*-poly(A) or the control vector CMV-IE-*miNeg-eGFP*-poly(A)) were subcloned into Ad5 gateway destination vectors for viral propagation and titering. HMVECs were transduced in normal growth medium and subjected to a 12-h wound healing assay. *A*, there was a significant increase in wound closure in cells transduced with Ad.CMV.*Irx3-v5* compared with the Ad.CMV.*Xnull-v5* control 6 h post-injury. Conversely, transduction with Ad.CMV.*mirIrx3-eGFP* significantly reduced wound closure 12 h post-injury compared with the Ad.CMV.*mirNeg-eGFP* control. *B*, temporal analysis of *Irx3*-induced wound closure 12 h post-injury. HMVECs transduced with Ad.CMV.*Irx3-v5* migrated and closed 100% of the original wound area compared with 58% wound closure in cells transduced with Ad.CMV-GFP-*mirIrx3-eGFP* and compared with the vector-matched controls Ad.CMV.*Xnull-v5* and Ad.CMV.*miNeg-eGFP. C*, representative gel image of *Irx3* mRNA expression in HMVECs after the indicated adenoviral vector transduction at 12 h. 18 S rRNA was used as an internal control. *D* and *E*, semiquantitative RT-PCR analysis of *Irx3* expression after transduction with the indicated adenoviral vector at 12 h. Data are expressed as -fold change *versus* the vector-matched controls (n ≥ 3). **, *p* < 0.01.

The effect of *Irx3* gain and loss of function on cell motility was assessed by wound healing assay (Figs. 2, *A* and *B*). HMVECs were transduced with recombinant adenoviral vectors containing a CMV immediate-early promoter (CMV-IE), human *Irx3* cDNA (BC023667.2)/tag on demand V5 epitope (Ad.CMV.*Irx3-v5* and Ad.CMV.*Xnull-v5*), CMV-IE/*mirIrx3*/*eGFP*, or synthetic scrambled control miRNA (Ad.CMV.*miIrx3-eGFP* and Ad.CMV.*miNeg-eGFP*) (m.o.i. = 20). Transduction of HMVECs with Ad.CMV.*Irx3-v5* resulted in increased wound healing as early as 6 h compared with the Ad.CMV.*Xnull-v5* control vector (Fig. 2*A*, *top panel*). In contrast, HMVECs transduced with Ad.*mirIrx3-eGFP* exhibited markedly reduced migration at 12 h compared with the negative control vector Ad.*mirNeg-eGFP* (Fig. 2*A*, *bottom panel*). Temporal assessment of the wound area in HMVECs, transduced with the Ad.*mirIrx3-eGFP*, indicate an area of 43.2 ± 5.46% at 12 h (Fig. 2*B*) *versus* control vector Ad.*mirNeg-eGFP*-transduced cells (95.6 ± 3.11%, *p* = 0.003). HMVECs transduced with the Ad.CMV.*Irx3-v5* vector had a remaining wound area of 24.7 ± 2.23% at 6 h and 92.8 ± 1.77% at 9 h (Fig. 2*B*) compared with cells transduced with the control Ad.CMV.*Xnull-v5* vector (52.1 ± 4.09% (*p* = 0.0073) and 28.4 ± 5.52% (*p* = 0.031), respectively).

To demonstrate effective gain or loss of function of *Irx3* expression, HMVECs were transduced with the indicated adenoviral vectors (m.o.i. = 20), and Irx3 mRNA levels were examined by quantitative RT-PCR (Fig. 2*C*). Our results in Fig. 2*D* indicate a significant -fold increase (29.45 ± 4.32, *p* = 0.0082) in Irx3 mRNA in cells transduced with Ad.CMV.*Irx3-v5* compared with the Ad.CMV.*Xnull-v5* control vector. In contrast, HMVECs transduced with Ad.CMV.*miIrx3-eGFP* (m.o.i. = 20) resulted in a significant -fold decrease (83.33 ± 6.24, *p* = 0.0031) in endogenous Irx3 mRNA (Fig. 2*E*) compared with cells transduced with the Ad.CMV.*miNeg-eGFP*) control vector. These data indicate that *Irx3* gain of function and loss of function can modulate wound closure rates in HMVECs and suggest a functional role of *Irx3* in mediating EC migration.

##### Irx3 Promotes Endothelial Cell Invasion and Chemotaxis

**FIGURE 3.**
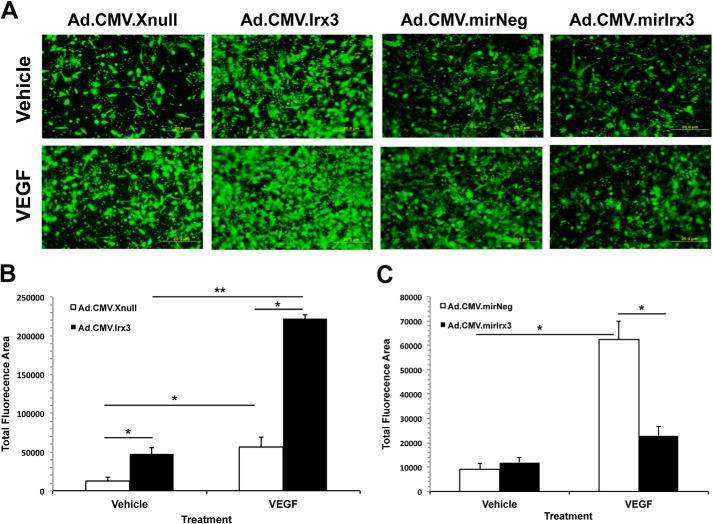
**Irx3 increases EC migration and invasion in a chemotactic VEGF gradient.** *A*, representative images of HMVECs transduced with the indicated adenoviral vectors (m.o.i. = 20) for 20 h and seeded in transwell migration plates precoated with fibronectin. VEGF-A (20 ng/ml) or vehicle was added to the lower chamber of the wells for 20 h and stained with CalceinAM dye for visualization. *B* and *C*, HMVECs transduced with Ad.CMV.*Irx3-v5* resulted in a significant increase in migration and invasion through the fibronectin-coated membrane (*B*), whereas HMVECs transduced with Ad.CMV.*mirIrx3-eGFP* (*C*) impeded migration compared with negative vector-matched controls. Magnification scale bar, 20 μm. Migration was quantified using ImagePro software (*n* ≥ 3). *, *p* < 0.05; **, *p* < 0.01.

The ability of endothelial cells to migrate and invade through the extracellular matrix in response to a VEGF-A gradient is essential for normal and pathophysiological angiogenesis *in vivo*. Therefore, we conducted transwell plate migration assays to determine whether *Irx3* expression accelerates HMVEC migration and invasion toward a chemoattractant *in vitro*. Minimal growth medium or medium supplemented with 20 ng/ml VEGF-A was added to the lower chambers for 20 h. Migration and invasion were quantified by measuring the amount of cells that migrated through the membrane (Fig. 3*A*) and are expressed in total fluorescence/well (number of pixels) in the lower chamber. Results from HMVECs transduced with Ad.CMV.*Irx3-v5* showed a total fluorescent area of 221,683 ± 12,861 pixels in response to VEGF-A treatment, whereas Ad.CMV.*Xnull-v5*-transduced cells displayed a significantly reduced area of 56,894 ± 3,178 (*p* = 0.015) pixels (Fig. 3*B*). Vehicle-treated cells transduced with Ad.CMV.*Irx3-v5* exhibited more migration and invasion, with a fluorescent area of 47,796 ± 5,954 pixels compared with cells transduced with Ad.CMV.*Xnull-v5* with an area of 12,930 ± 2066 pixels (*p* = 0.043). HMVECs transduced with Ad.*mirIrx3-eGFP* had a fluorescent area of 11,943 ± 2869 pixels compared with cells transduced with Ad.*mirNeg-eGFP*, which had a significantly larger area of 62,369 ± 4251 pixels (*p* = 0.014) in response to VEGF-A (Fig. 3*C*). No significant difference was observed in the migration of transduced and vehicle-treated cell fluorescent areas (Ad.*mirNeg-eGFP*, 9161 ± 4347 pixels; Ad.*mirIrx3-eGFP*, 11,974 ± 2896 pixels). These results suggest that *Irx3* promotes chemotactic migration and invasion of ECs in response to VEGF stimulation.

##### Irx3 Increases Endothelial Tube-like Formation and Network Complexity in Vitro

**FIGURE 4.**
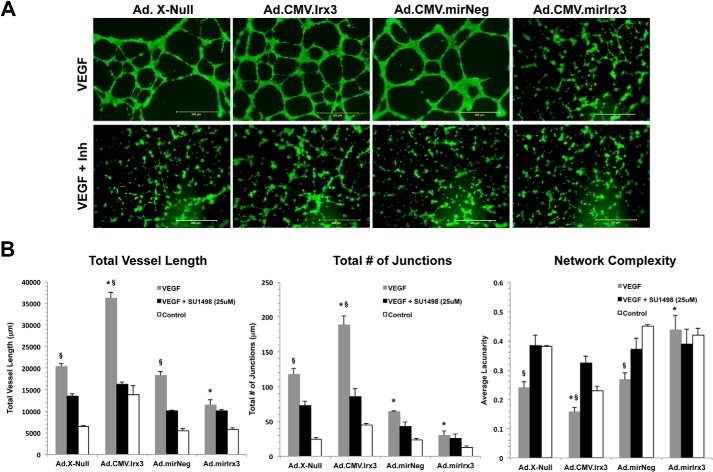
**Irx3 increases EC tube-like network formation.** HMVECs were transduced with the indicated adenoviral vector (m.o.i. = 20) for 12 h and seeded at density of 2 × 10^5^ cells/well of 96-well Matrigel-coated plates with VEGF-A (20 ng/ml) for 18 h. The inhibitor SU1498 (25 μm) was preincubated prior to VEGF treatment for 2 h under the indicated conditions. Cells were then stained with CalceinAM for visualization. *A*, representative images of vector-transduced capillary-like networks. *B*, quantitative analysis of total vessel length, number of junctions, and vascular network complexity (lacunarity) was performed using NIH AngioTool software (*n* ≥ 3). *, *p* < 0.05 compared with vector-matched control; §, *p* < 0.05 compared with SU1498 inhibition within vector groups. *Scale bars* = 300 μm.

To examine the effects of *Irx3* on tube formation by HMVECs, cells were transduced with either Ad.CMV.*Irx3-v5*, Ad.CMV.*Xnull-v5*, Ad.*mirIrx3-eGFP*, *or* Ad.*mirNeg-eGFP* (m.o.i. = 20) for 18 h. As shown in Fig. 4, *A* and *B*, cells transduced with Ad.CMV.*Irx3-v5* had a total vessel length of 14,155 ± 3489 μm, which was significantly higher than the total vessel length of the Ad.*mirIrx3-eGFP* cells (5350 ± 857 μm, *p* = 0.038). Cells transduced with Ad.CMV.*Irx3-v5* exhibited a significantly higher number of cellular junctions (48 ± 5) compared with the Ad.CMV.*Xnull-v5* control (26 ± 3, *p* = 0.031). Transient knockdown of *Irx3* in HMVECs transduced with Ad.*mirIrx3-eGFP* resulted in the lowest number of junctions (12 ± 2), whereas the Ad.*mirNeg-eGFP* transductants had 29 ± 2 junctions (*p* = 0.044). We observed a significant increase in vessel complexity in response to *Irx3*-transduced cells. Complexity was measured using a computational algorithm available through the AngioTool software. The results demonstrate a significant decrease in network complexity in Ad.CMV.*Irx3-v5*-transduced HMVECs (lacunarity, 0.23 ± 0.04) compared with Ad.CMV.*Xnull-v5*-transduced cells (lacunarity, 0.38 ± 0.02). No significant difference was seen in cells transduced with either Ad.*mirIrx3-eGFP* (lacunarity, 0.43 ± 0.04) or the Ad.*mirNeg-eGFP* control condition (lacunarity, 0.46 ± 0.01).

Next, cells were transduced with viral vectors and treated with VEGF for 18 h. Capillary-like networks were examined. The results show that Ad.CMV.*Irx3-v5*-transduced cells had an increase in the number of junctions (190 ± 11), which were significantly more numerous than in Ad.CMV.*Xnull-v5* cells (116 ± 8, *p* = 0.045). Cells transduced with Ad.*mirIrx3-eGFP* had a significant reduction in junctions (28 ± 5) compared with the Ad.*mirNeg-eGFP* control vector (69 ± 4, *p* = 0.031). Total vessel length was also increased, with the longest total length in Ad.CMV.*Irx3-v5*-transduced cells (36,154 ± 2034 μm) compared with Ad.CMV.*Xnull-v5* control cells (20,120 ± 879 μm, *p* = 0.039). Interestingly, we found that the addition of VEGF had only a moderate impact on network complexity in cells transduced with Ad.CMV.*Irx3-v5* (0.16 ± 0.05) *versus* cells transduced with Ad.*mirIrx3-eGFP* (0.46 ± 0.06, *p* = 0.048). To determine whether the observed effects were VEGF-dependent, we treated cells with the VEGFR2 inhibitor SU1498 (25 μm). Interestingly, our results show that SU1498 significantly reduced network vessel length, the number of junctions, and network complexity under most conditions except in HMVECs transduced with Ad.*mirIrx3-eGFP*. These results indicate that *Irx3* modulates tube-like capillary networks in a VEGF-dependent manner.

##### Irx3 Increases the Percentage of the Dll4^+^ EC tip Cell Marker Population

**FIGURE 5.**
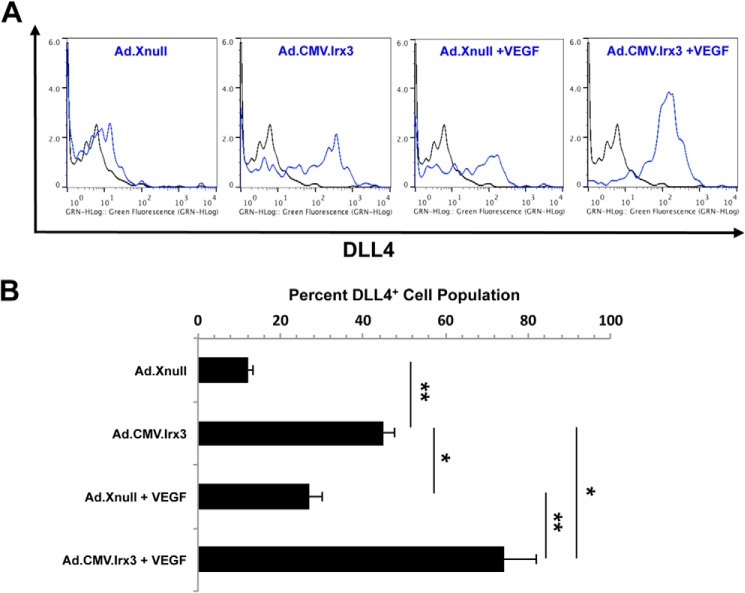
**Irx3 increases the percentage of the Dll4^+^ microvascular endothelial cell population.** Fluorescence microcytometry analysis of HMVECs following wound induction and VEGF-A (20 ng/ml) or vehicle treatment for 12 h demonstrates an increase in the expression of the EC tip cell fate marker DLL4 in cells transduced with Ad.CMV.*Irx3-v5* compared with the control vector. *A*, representative microcytometry histogram images of HMVECs transduced with the indicated vector for 24 h and subjected to wound healing assay with or without VEGF-A treatment for 9 h. Cells were fixed and stained with anti-DLL4 (1:400, *blue line*) or isotype control (IgG2a, 1:600, *black line*) antibodies with Alexa Fluor 488-conjugated anti-mouse secondary for visualization. A total of 30,000 events/well were measured (3 × 10,000 counts) and analyzed using FlowJo software. *B*, quantitative analysis of the DLL4^+^ EC population in response to transduction of the indicated vector and VEGF-A treatment (*n* ≥ 3). *, *p* < 0.05; **, *p* < 0.01.

We postulated that *Irx3* may be mediating the observed phenotypes by increasing EC tip cell fate specification, resulting in the observed increase in EC migration, invasion, and EC tube-like network complexity. Therefore, we performed fluorescence microcytometry studies on cells transduced with Ad.CMV.*Irx3-v5*, Ad.CMV.*Xnull-v5*, Ad.*mirIrx3-eGFP*, or Ad.*mirNeg-eGFP* (m.o.i. = 20). Multiple scratch wounds were made on a rectangular cell culture plate, followed by 9-h treatment with VEGF or vehicle. Cells were collected and stained with an anti-DLL4 monoclonal antibody or an IgG2b-negative control, and positive counts (30,000 events/well) were collected and compared with IgG2b (Fig. 5*A*). Our results, under vehicle conditions, show that cells transduced with Ad.CMV.*Irx3-v5* contain a higher population of DLL4^+^ cells (42.8 ± 3.3%) *versus* Ad.CMV.*Xnull-v5*-transduced cells (16.2 ± 2.8, *p* = 0.0083). The results from cells treated with VEGF indicate that 74.8 ± 6.2% of cells in the Ad.CMV.*Irx3-v5*-transduced cell population were DLL4^+^ compared with Ad.CMV.*Xnull-v5*-transduced cells (23.6 ± 3.8%, *p* = 0.0092) (Fig. 5*B*). Of interest is the observation that Ad.CMV.*Irx3-v5* vehicle-treated cells contained a significantly higher population of DLL4^+^ cells *versus* cells transduced with Ad.CMV.*Xnull-v5* and VEGF (*p* = 0.047). These results suggest that the expression of *Irx3* correlates with an increase in the DLL4^+^ cell population, which is increased further synergistically upon VEGF treatment.

##### The Notch Mediator HEY1 Is a Negative Regulator of Irx3

**FIGURE 6.**
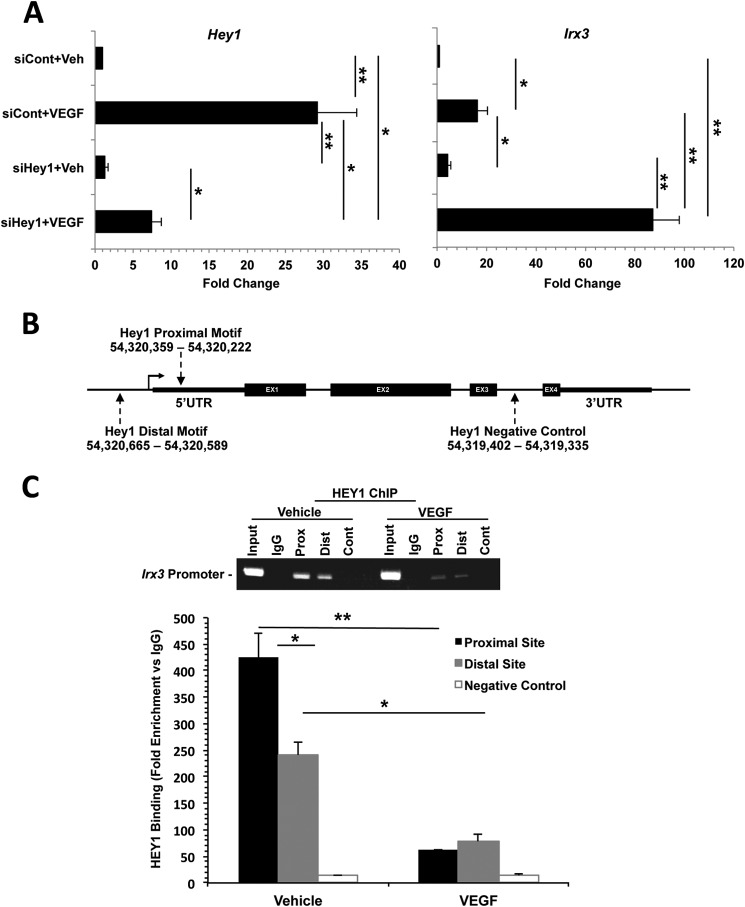
**HEY1 is a negative regulator of *Irx3* and binds to the Irx3 promoter.** *A*, quantitative RT-PCR validation results for the high-throughput siRNA screen, showing a significant increase in *Irx3* expression in HMVECs following knockdown of *Hey1* with or without VEGF-A for 12 h compared with negative control siRNA (*siCont*) treatment conditions. *Veh*, vehicle. *B*, schematic of the predicted ENCODE HEY1 binding sites on *Irx3* regulatory regions. The HEY1 proximal (chr16: 54,320,359–54,320,222) and distal (chr16: 54,320,685–54,320,589) binding sites are shown relative to the transcriptional start site. Primers were designed within an intronic region of *Irx3* as a negative control. *C*, Quantitative analysis of HEY1 binding on Irx3 promoter region. Representative PCR gel image of enriched chromatin product at the indicated sites with or without VEGF treatment. *Prox*, proximal; *Dist*, distal. Quantitative PCR of enriched chromatin around the indicated HEY1 binding sites. Values are expressed as -fold enrichment *versus* IgG control input. Chromatin from HMVECs at 12 h with or without VEGF was precipitated with an anti-HEY1 antibody (1:200) or IgG (1:400). DNA was recovered from immunoprecipitated chromatin and total input chromatin and analyzed by real-time PCR. Primer pairs used for quantitative PCR were specific to either the proximal, distal, or negative control intronic regions. A significant reduction in HEY1 binding was observed in response to VEGF treatment compared with controls (n ≥ 3). *, *p* < 0.05; *, *p* < 0.01.

We utilized a high-throughput siRNA screen (supplemental Table 1) to identify potential negative and positive regulators of *Irx3* in response to 12 h of VEGF treatment. HMVECs were reverse-transfected in multiwell plates containing two validated siRNAs for a single transcription factor. Next, cells were treated with VEGF for 12 h, total RNA was harvested, and quantitative RT-PCR was performed. Analysis of the high-throughput siRNA screen revealed an 87.3- ± 10.7-fold (*p* = 0.0034) increase in *Irx3* expression upon siRNA silencing of *Hey1* compared with vehicle-treated negative siRNA control wells or negative siRNA control wells treated with VEGF (6.15- ± 0.82-fold; *p* = 0.0086). To confirm siRNA silencing of *Hey1*, we performed quantitative RT-PCR in the *Hey1* siRNA-targeted well and compared the results with the negative siRNA control wells (Fig. 6*A*). Our results confirm that *Hey1* mRNA is reduced significantly reduced in wells containing siRNA targeted to *Hey1* (by 78.3 ± 6.73%, *p* = 0.021) compared with VEGF-treated siRNA negative control conditions. Silencing of *Hey1* under vehicle conditions was not significant compared with siRNA vehicle control conditions. Therefore, these data suggest that the downstream Notch signaling transcription factor HEY1 is a negative regulator of *Irx3* expression during VEGF treatment, and it has been shown in previous studies to play a critical role in the regulation of developmental and pathophysiological angiogenesis (10).

##### The Notch Downstream Transcription Mediator HEY1 Directly Binds the Irx3 Promoter in Vivo

HEY bHLH proteins are well known to function as transcriptional repressors during angiogenesis by binding a class B E-box consensus motif (5′-CACGYG-3′) (47). We identified two putative binding HEY1 binding sites in the ENCODE (Encyclopedia Of DNA Elements) transcription factor ChIP-seq track on the University of California at Santa Cruz genome browser: GRCh37.1/hg19 assembly, located at chr16:54320222–54320359 (proximal 5′-*CACGTG*-3′), and chr16:54320589–54320665 (distal 5′-GCGCCG-3′) within 1 kb of the transcriptional start site. To confirm whether HEY1 binds the *Irx3* promoter directly or through an intermediate cofactor, we performed ChIP experiments to determine HEY1 binding. HMVECs were harvested after 12 h with or without VEGF treatment and subjected to protein-DNA cross-linking. After precipitation with anti-HEY1 or an IgG2 isotype control, DNA fragments were reverse-cross-linked and PCR-amplified with primers sets flanking the predicted proximal or distal HEY1 binding sites in the upstream regulatory region of the *Irx3* promoter. Primers designed to amplify an intronic region between exons 3 and 4 of *Irx3* were used as a negative control (Fig. 6*B*). The results in Fig. 6*C* indicate that, at 12 h under vehicle conditions, chromatin-fold enrichment at the proximal HEY1 binding site was 424.43- ± 32.71-fold and 240.98- ± 28.6-fold (*p* = 0.038) at the proximal and distal binding sites, respectively. Cells treated for 12 h with VEGF resulted in alleviated HEY1 binding of the *Irx3* promoter with significantly less enrichment (73.57- ± 6.82-fold) at the proximal HEY1 binding site (*p* = 0.0028) and 77.56- ± 23.02-fold enrichment at the distal site (*p* = 0.034) compared with vehicle controls. The intronic region of *Irx3* showed no significant change in -fold enrichment under either condition. Taken together, these results indicate that the Notch signaling mediator HEY1 is a negative regulator of *Irx3* in the absence of VEGF and binds directly to the *Irx3* promoter. Furthermore, stimulation with VEGF ligand partially alleviates HEY1-mediated repression of *Irx3* in HMVECs.

## DISCUSSION

Angiogenesis is a complex and carefully orchestrated series of molecular and cellular events requiring both the proliferation and migration of endothelial cells (29, 30). Although there are numerous studies characterizing the signal transduction pathways essential for angiogenesis, greater insight is needed into transcription regulatory networks that govern EC migration and fate specification (31–33). Homeobox genes are characterized as early mediators of embryonic developmental patterning (34, 35). Indeed, *Irx3* expression has been observed in early developmental stages, which indicates that *Irx3* is an early mediator of transcription signaling to regulate downstream gene targets for normal embryogenesis (36, 37). Genetic lineage mapping studies by others, using the endothelial lineage-specific marker *Tie2::Cre* in the mouse heart, suggested functional significance of both *Irx3* and *Irx5* in PECAM^+^ (platelet/endothelial cell adhesion molecule 1) cells as early as embryonic day 14 (13). Therefore, we investigated the temporal expression of *Irx3* in endothelial cells in response to VEGF stimulation and in *in vivo* rat carotid arteries. Our findings indicate that *Irx3* is expressed in HMVECs, that it is temporally regulated by VEGF as early as 8 h after VEGF-A treatment, and that it is maintained over a period of 48 h. Our *in situ* hybridization studies demonstrate expression in adult rat carotid arterial endothelium that colocalized with cells coexpressing *Pecam1*. Of note, we also observed *Irx3* expression in the arterial medial layer, suggesting expression in vascular smooth muscle cells. Although previous mouse studies in the heart indicate coexpression of *Irx3* and *Irx5* in the endocardium, we did not detect expression of *Irx5* in HMVECs under quiescence or in response to VEGF stimulation in our studies (data not shown). This may be due to the unique requirement of *Irx5* to regulate *Kcnd2* expression to establish a polarizing potassium gradient between the endo- and epicardial cushion layers (38). Therefore, our observations are somewhat in agreement with these previous studies. However, greater insight is needed to identify downstream *Irx3* transcription targets in the endothelium of the peripheral vasculature and perform metadata analyses from other organ systems to decipher common and specific gene targets of IRX proteins in a stringent manner.

Early reports in *Drosophila* describe the requirement of the IRO proteins *Araucan* (*Ara*) and Caupolican (*Caup*) for proper wing vein formation. Furthermore, genetic disruption of the *Ara* and *Caup* loci results in the loss of L1-L5 wing vein formation (17, 39). Our studies demonstrated that transient knockdown of *Irx3* in HMVECs suppressed VEGF-induced cell migration during wound closure and chemotactic migration, whereas enforced expression of *Irx3* resulted in accelerated migration in wound closure and chemotaxis. The results of the chemotaxis assays are particularly interesting because directional migration is critical to embryonic vessel formation, but the transcription regulatory mechanism(s) that enable endothelial cells to respond to chemotactic factors such as VEGF are not fully understood (40). Here we show that *Irx3* is a proangiogenic mediator and that it functions to promote endothelial cell migration, which is essential to productive angiogenesis. These observations highlight the possibility that Ara- and Caup-mediated *Drosophila* wing vein formation abnormalities may also be due, in part, to the reduced migratory potential of cells that line the vein. Indeed, studies performed in *Xenopus laevis* suggest that *Irx5* functions to orchestrate migration by repressing cranial neural crest and primordial progenitor cell populations by repressing stromal cell-derived factor-1 (*SDF-1/CXCL12*) expression (41).

The concerted activation of Notch and VEGF signaling is essential to maintain stable vessel networks during embryonic and postnatal angiogenesis (42–45). During sprouting angiogenesis, new vessels are led by migrating endothelial tip cells, characterized as non-proliferating migratory cells that express higher levels DLL4 and VEGFR2 surface protein markers (46). Endothelial tip cell fate selection is governed by VEGF ligand binding, whereas DLL4/Notch signaling activation in neighboring cells specifies a proliferative, non-migrating EC stalk cell fate by lateral inhibition, although the complete process remains to be fully defined (46). Our gain of function studies demonstrate that *Irx3* increases vascular junction number, total vessel length, and vessel network complexity, whereas silencing of endogenous *Irx3* resulted in the opposite effect. Moreover, our data demonstrate a significantly higher percentage of Dll4^+^ HMVECs, a marker of the EC tip cell phenotype in *Irx3* gain of function experiments. The VEGFR2 inhibitor SU1498 partially blocked the effect of enforced *Irx3* expression, which suggests a partial VEGF signaling dependence. Surprisingly, the VEGF kinase inhibitor SU1498 had no noticeable effect on network total length, junction number, and complexity under *Irx3* silencing conditions. This is most likely due to a significant loss of endogenous Irx3, resulting in reduced HMVEC migration, invasion, and capillary-like network formation.

In the context of angiogenesis, HEY proteins are known to be downstream transcription repressors of Notch signaling to repress VEGFR-2 expression in proliferative stalk ECs (47). Our siRNA screening studies identified HEY1 as a repressor of *Irx3* expression in response to VEGF treatment. VEGFR-2 expression in ECs is required for promoting migration and proliferation during angiogenesis. Genetic or pharmacological manipulation of either Notch signaling activity or HEY1 expression can severely disrupt EC angiogenesis in *in vitro* tubulogenesis assays and in *Hey1*^−/−^*Hey2*^−/−^ null mice (46, 48). It is also important to note that HEY1 does not bind to VEGFR-2 via canonical E-box motifs but, rather, through multiple weak interactions with specificity proteins on GC-rich sequences upstream of a transcription initiator element (27, 47).

Our ChIP studies confirmed the results of our siRNA screen, demonstrating that HEY1 binds to the *Irx3* promoter at a proximal binding site and a distal binding site with increased -fold enrichment in the absence of VEGF. Interestingly, stronger binding was observed at the proximal site that consisted of a GC-rich region downstream of the *Irx3* transcription initiator sequence, whereas weaker binding was observed at the classical bHLH HEY1 binding site located in the upstream regulatory region. Occupancy of the *Irx3* promoter by HEY1 is consistent with HEY1-mediated repression of genes, resulting in a quiescent state in endothelial cells, and repression is alleviated by VEGF-A treatment (27, 49). This correlates to a decrease in *Irx3* mRNA expression when cultured HMVECs become confluent 48 h after VEGF-A treatment. Our data suggest that HEY1 may be binding to the *Irx3* promoter by a similar mechanism as HEY1 binding to VEGFR-2, which may involve GC-rich, SP1-dependent cofactor association of HEY1 on the *Irx3* proximal region 5′-UTR region. Future experiments to stringently dissect the mechanism through which Notch signaling and HEY1 directly interact with the *Irx3* promoter and whether it requires interactions with transcription cofactors, such as SP1 proteins, are required.

**FIGURE 7.**
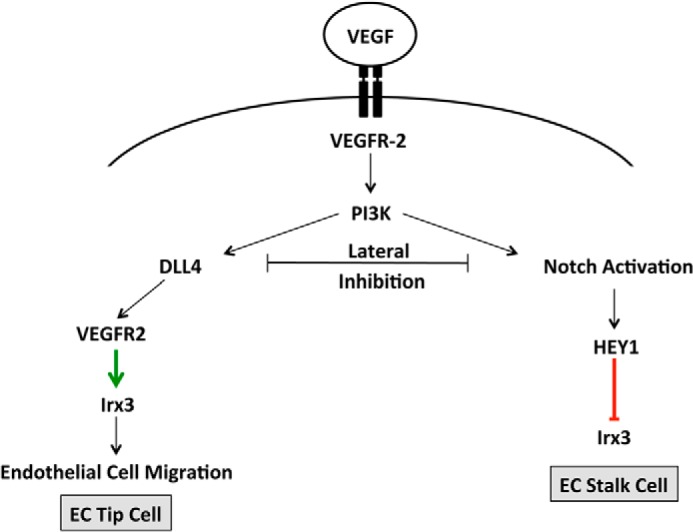
**Schematic of *Irx3* in response to VEGF and Notch signaling in HMVECs.** VEGF-A binds preferentially to VEGFR-2, initiating dimerization of the receptor inducing Notch/Dll4 activation. Notch-mediated lateral inhibition results in increased Dll4 expression and EC tip cell fate and Notch signaling activation, specifying EC stalk cell fate, where HEY1 functions to maintain repression of *Irx3*, resulting in a non-migratory phenotype. In EC tip cells, Dll4 expression leads to an up-regulation of VEGFR-2 transcription and, subsequently, *Irx3* expression. In response to an increase in VEGFR-2-mediated *Dll4* expression, HEY1 is down-regulated and results in derepression of *Irx3* and promotion of increased EC migration and capillary-like vascular network complexity.

In summary, our findings describe a novel functional role of Irx3 as a proangiogenic mediator of VEGF and Notch signaling by promoting EC migration and EC tip cell fate specification in response to VEGF stimulation (Fig. 7). Reports indicate that *Irx3* null mice are viable, although they exhibit multiple phenotypic abnormalities that become more severe under pathological conditions (50). The peripheral vasculature of *Irx3* and *Irx3/Irx5* null mice should be examined in the context of adult angiogenesis models for further insights into the role of *Irx3 in vivo* and to see whether any *Irx* family functional redundancy exists. In the context of angiogenesis, Notch activation by DLL4 results in the cleavage of the Notch intracellular domain, its association with CBF1, and the subsequent up-regulation of target genes such as *Hey1* (9, 43). Clinical studies have shown only moderate success in cancer therapy with VEGF receptor inhibitors (51). Emerging new approaches that target the Notch signaling ligand DLL4 result in excessive non-productive angiogenesis, affecting both tumor growth and metastatic behavior in tumors that are refractive to anti-VEGF therapies, although off-target effects are possible (52). In light of these studies, more targeted therapeutic approaches, such as miRNAs to downstream factors of VEGF and/or Notch signaling, should be explored to minimize adverse complications. Indeed, pharmacological approaches to regulate IRX3 expression and function during adult angiogenesis, in the context disease-induced ischemia, may provide exciting new therapeutic opportunities.

## Footnotes

2 The abbreviations used are:

EC: endothelial cell
HMVEC: human dermal microvascular endothelial cell
eGFP: enhanced GFP
m.o.i.: multiplicity of infection
IE: immediate-early
VE: vascular endothelium
HES: hairy enhancer of split
HEY/HESR: hes-related transcription factor with YRPW motif.
